# Effects of continuous hypoxia on flow-mediated dilation in the cerebral and systemic circulation: on the regulatory significance of shear rate phenotype

**DOI:** 10.1186/s12576-022-00841-5

**Published:** 2022-07-20

**Authors:** Shigehiko Ogoh, Takuro Washio, Benjamin S. Stacey, Hayato Tsukamoto, Angelo Iannetelli, Thomas S. Owens, Thomas A. Calverley, Lewis Fall, Christopher J. Marley, Damian M. Bailey

**Affiliations:** 1 Department of Biomedical Engineering, Toyo University, Kawagoe, Saitama Japan; 2 Neurovascular Research Laboratory, Faculty of Life Sciences and Education, University of South Wales, Pontypridd, CF37 4AT UK; 3 Research Fellow of Japan Society for the Promotion of Science, Tokyo, Japan; 4 Faculty of Sport and Health Science, Ritsumeikan University, Shiga, Japan

**Keywords:** Hypoxia, Cerebral blood flow, Flow-mediated dilation, Endothelial function, Antegrade shear rate, Retrograde shear rate

## Abstract

Emergent evidence suggests that cyclic intermittent hypoxia increases cerebral arterial shear rate and endothelial function, whereas continuous exposure decreases anterior cerebral oxygen (O_2_) delivery. To examine to what extent continuous hypoxia impacts cerebral shear rate, cerebral endothelial function, and consequent cerebral O_2_ delivery (CDO_2_), eight healthy males were randomly assigned single-blind to 7 h passive exposure to both normoxia (21% O_2_) and hypoxia (12% O_2_). Blood flow in the brachial and internal carotid arteries were determined using Duplex ultrasound and included the combined assessment of systemic and cerebral endothelium-dependent flow-mediated dilatation. Systemic (brachial artery) flow-mediated dilatation was consistently lower during hypoxia (*P* = 0.013 *vs*. normoxia), whereas cerebral flow-mediated dilation remained preserved (*P* = 0.927 *vs*. normoxia) despite a reduction in internal carotid artery antegrade shear rate (*P* = 0.002 *vs*. normoxia) and CDO_2_ (*P* < 0.001 *vs*. normoxia). Collectively, these findings indicate that the reduction in CDO_2_ appears to be independent of cerebral endothelial function and contrasts with that observed during cyclic intermittent hypoxia, highlighting the regulatory importance of (hypoxia) dose duration and flow/shear rate phenotype.

## Introduction

In peripheral conduit arteries, it is well established that an increase in antegrade shear rate (SR) stimulated by an acute elevation in blood flow improves systemic vascular endothelium-dependent vasodilatory function [1–3]. This provides the hemodynamic basis underlying the vascular protective benefits of physical exercise to improve systemic endothelial function (EF) and decrease the risk of cardiovascular disease [4]. Equally, cerebrovascular endothelial dysfunction predisposes to stroke and neurodegenerative diseases [5, 6] and can be countered by flow-mediated elevations in SR [6].

Recently, cyclic intermittent hypoxia, consisting of 3–10 bouts of intermittent exposures (3–6 min) to moderate hypoxia (10–15% O_2_), was shown to improve cerebral EF subsequent to cerebral blood flow (CBF)-mediated sinusoidal elevations in cerebral SR, implying that intermittent hypoxia may be a useful non-pharmacological adjunct to optimize cerebrovascular health [7]. In further support, cyclic intermittent exercise increases cerebral SR more effectively than continuous steady-state exercise [8]. Therefore, the improvement of EF may be dictated or subject to regulation by the specific flow/SR ‘phenotype’. Given that continuous exposure to hypoxia is also useful as a clinical therapy [9, 10], further mechanistic investigation is required. To what extent, if indeed any, continuous steady-state exposure to hypoxia, a stimulus defined by an entirely different hemodynamic phenotype (i.e., non-cyclic/sinusoidal), impacts cerebral SR and consequent EF.

Our recent study [11] demonstrated that CDO_2_ decreased in the anterior cerebral circulation during continuous exposure (7 h) to hypoxia, indicating that steady-state exposure, unlike its cyclic intermittent counterpart, attenuates cerebral bioenergetic function. The physiological mechanisms underlying these divergent findings remain to be examined. Furthermore, there are no integrated studies to the best of our knowledge that have simultaneously examined changes in both local (cerebral) and systemic (brachial) EF during continuous hypoxia. This is surprising, given the controversial findings relating to systemic flow-mediated dilation (FMD) in hypoxia [12–22] combined with the observation that retrograde flow is confined to the systemic and not the cerebral arterial circulation [11, 23, 24]. Specifically, the increase in retrograde flow [22, 25, 26], known to attenuate systemic EF [27], given its absence in the cerebral circulation [8, 23, 24, 28] would result in preserved (i.e., maintained) EF.

In light of these knowledge gaps, we conducted a randomized, cross-over, single-blind trial in normoxia and hypoxia to examine to what extent continuous steady-state exposure to inspiratory hypoxia affects the integrated CBF-mediated regulation of cerebral and systemic SR and consequent EF. We hypothesized that unlike cyclic intermittent exposure, continuous steady-state exposure to hypoxia would not alter cerebral SR or EF, and that the cerebral FMD response to continuous hypoxia would differ from that observed in the systemic circulation.

## Materials and methods

### Participants

Eight physically active males (age: 23 ± 2 y, stature: 1.81 ± 0.04 m, mass: 80 ± 7 kg) were recruited from the local University student population by word-of-mouth, social media platforms and advertisements. All participants lived close to sea level (90 m) and had not been exposed to simulated or terrestrial high-altitude in the previous 12 months. Following a medical examination, they were confirmed to be healthy and free of any known diseases. Furthermore, they were not taking any prescribed or over-the-counter medications or supplements. They were instructed to refrain from physical activity, caffeine, and alcohol and to follow a low nitrate/nitrite diet 24 h prior to formal experimentation [29].

### Design

**Fig. 1:**
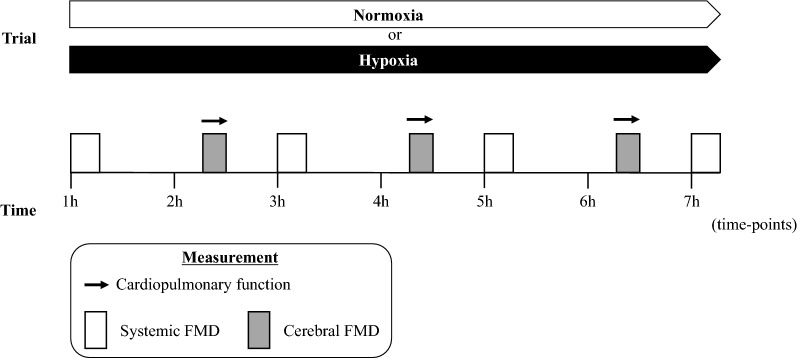
Experimental protocol

The present study adopted a randomized, cross-over, single-blind design with select measurements (see below) performed throughout (Fig. 1). Participants completed two different experimental trials in a normobaric environmental chamber (~ 120 m^3^) maintained at 21 °C and 50% relative humidity (Design Environmental, Ebbw Vale, UK). They were randomly assigned single-blind to complete 7 h passive exposure to normoxia (FiO_2_ = 0.21) and 7 h of normobaric hypoxia (FiO_2_ = 0.12), separated by 7 days. Participants arrived at the laboratory (between 8:00 and 9:00 A.M.) following a 12 h overnight fast and consumed a standardized meal (30 g of oats with 180 mL water), 30 min before the experimental trials. They received the standardized meal again at 2 h, 4 h, and 6 h to maximize compliance and avoid hunger/dehydration [30]. Flow-mediated dilation (FMD), as an index of systemic vascular EF, of the BA (systemic FMD) and FMD, as an index of cerebral EF, of the internal carotid artery (ICA, cerebral FMD) were determined every 2 h from the first (systemic FMD, 4 repeat measurements) or second hour (cerebral FMD, 3 repeat measurements) of experimentation (Fig. 1).

### Measurements

#### Cardiopulmonary function

Heart rate (HR) was monitored by ECG (lead II) and beat-to-beat arterial blood pressure (ABP) was monitored continuously via finger photoplethysmography (Finometer PRO, Finapres Medical Systems, Amsterdam, The Netherlands). SpO_2_ was quantified via finger-pulse oximetry (WristOx2^®^ 3150, Nonin, Minnesota, USA). The finometer blood pressure waveform was used to calculate mean arterial blood pressure (MAP) after calibrating values to the average of two automated brachial blood pressure measurements (Life Source, A&D Medical, model: UA767FAM), taken over a 5-min resting baseline period. The end-tidal partial pressure of carbon dioxide (P_ET_CO_2_) was measured via a mouthpiece and an automatic breath-by-breath respiratory gas-analyzing system consisting of a differential pressure transducer, sampling tube, filter, suction pump, and mass spectrometer (ML 206, ADInstruments, UK). All data were recorded continuously at 1 kHz.

#### Hemodynamic function

Diameter and blood velocity in the BA and ICA were determined using Duplex ultrasound (BA, Terason *t*3000, ICA; Vivid-i, GE Medical Systems, Tokyo, Japan). BA measurements were performed in the longitudinal section ~ 3–5 cm above the antecubital fossa. ICA measurements were performed ~ 1.0–1.5 cm distal to the carotid bifurcation with the participant’s chin slightly elevated. The steering angle was fixed to 60° and the sample volume was placed in the center of the vessel adjusted to cover the entire vascular lumen. We captured arterial images and associated velocity waveforms at 30 Hz and stored them in a computer for the subsequent assessment of systemic and cerebral FMD (see below).

#### Systemic FMD

BA FMD (as systemic FMD) was determined as the percent change in peak BA diameter during ischemia-induced reactive hyperemia from (pre-ischemic) resting control baseline according to established methods [31]. We used an inflation/deflation pneumatic cuff to provide the ischemic stimulus. Specifically, we recorded baseline scans assessing resting vessel diameter and velocity over 1 min. We then inflated the cuff to > 220 mmHg for 5 min. We resumed diameter and blood velocity recordings 30 s before cuff deflation and continued recording for 3 min thereafter.

#### Cerebral FMD

ICA FMD (as cerebral FMD) was determined as the percent change in peak ICA diameter during hypercapnia-induced reactive hyperemia [7, 32, 33]. We recorded a 1 min baseline period followed by 3 min of breathing 5% CO_2_ in 21% O_2_ (normoxic trial) or 12% O_2_ (hypoxic trial) with balanced nitrogen (N_2_) balance from a 200L Douglas Bag via Falconia tubing (Cranleigh, UK) connected to the inspiratory port of a two-way nonrebreathing valve (Hans Rudolph, 2400 series).

### Data analysis

HR, ABP and P_ET_CO_2_ were continuously measured throughout and averaged over 30 s every 2 h and complemented by the assessment of cerebral FMD. BA and ICA SR and FMD were analyzed during the hyperemic challenge (see above).

For systemic FMD, BA diameter and mean blood velocity during the FMD test were assessed at 30 Hz using custom-designed edge-detection and wall-tracking software (Blood Flow Analysis, Version 5.1). BA parameters were derived using an algorithm reported previously [34, 35]. One minute of baseline data were analysed to yield median baseline diameter (D_base_), peak (D_peak_), and time-to-peak diameter, blood flow and SR characteristics [34, 36].

In the cerebral FMD, similarly with systemic FMD, ICA diameter and blood velocity were analyzed at 30 Hz using custom-designed edge-detection and wall-tracking software (version 2.0.1, S-13037, Takei Kiki Kogyo, Tokyo, Japan). We interpolated ICA parameters to 1 Hz prior to subjecting data to a two-stage filtering process (median filter and Savitzky–Golay finite impulse response smoothing filter). In brief, (D_base_) and SR were analysed during the last minute and peak diameter (D_peak_) were then assessed visually to ensure that: the detected peak SR preceded the detected D_peak_ [7, 31–34]. BA and ICA SR were calculated using the equation; 4 × mean velocity/arterial diameter. In addition, the SR area under the curve (SR_AUC_) was calculated for data up to the point of D_peak_ using the trapezoid rule [3, 36]. FMD was calculated using peak and baseline values [(D_peak_–D_base_)/D_base_ × 100]. Normalized FMD was calculated by dividing FMD by the SR_AUC_.

Cerebral oxygen delivery (CDO_2_) were determined as CDO_2_ (mL/min)  = ICA blood flow × (estimated) arterial O_2_ content (CaO_2_), calculated as $\left(\mathrm{Hb }(\mathrm{g}/\mathrm{dL})\times 1.39 \times \frac{{\mathrm{SaO}}_{2 \text{(\%)} }}{100}\right)$ excluding (albeit minor) contributions from dissolved O_2_ (0.003 × arterial PO_2_), since we did not perform arterial catheterization.

### Statistical analysis

#### Power calculation

Data were analyzed using G^∗^ Power 3.1 software. Assuming a comparable hypoxia-induced reduction (14%) and corresponding effect size (*η*^2^ = 1.267) for brachial (systemic) FMD previously observed by our group in a similar demographic [15], the present study required a (minimum) sample size of 6 participants (within groups, repeated measures) to achieve a (minimum) power of 0.80 at *p* < 0.05. We chose to further inflate this to *n* = 8 during recruitment given the potential for loss to follow-up owing to technical failure/drop-out. We were not in a position to prospectively power against cerebral FMD given that this was the first study to investigate this metric.

#### Inferential statistics

All data were analyzed using SPSS (IBM SPSS Statistics Version 28.0) and expressed as mean ± standard deviation (SD). A linear mixed model with fixed effects for *Trial* (normoxia vs. hypoxia) or *Inspirate* (eucapnia vs. hypercapnia) and *Time* (0–7 h) was used to compare acquired data. In addition, the change in hypercapnia on cardiopulmonary data from eupnea to hypercapnia during cerebral FMD assessment (i.e., ΔHR, MAP and P_ET_CO_2_) were evaluated using a linear mixed model. To identify the effect of D_base_, corrected systemic and cerebral FMD were calculated by using D_base_ as covariates [37, 38]. Differences between means were located using Bonferroni-corrected paired samples *t* tests. Pearson correlation was used to analyze the statistical relationship between CDO_2_ and cerebral FMD. Significance was determined at an alpha level of 0.05 for all two-tailed tests.

## Results

### Loss to follow-up

Systemic FMD was determined in all participants, whereas ICA velocity and/or diameter data were lost in 2 or 3 participants during hypercapnia. Thus, the cerebral FMD sample size reflects data obtained in 6 participants except for 4 h normoxia and 6 h hypoxia (*n* = 5, Fig. 3).

### Cardiopulmonary function

MAP and P_ET_CO_2_ were lower in hypoxia (*P* < 0.001 *vs*. normoxia), whereas HR was higher (*P* = 0.001 *vs*. normoxia). The hypercapnia stimulation during cerebral FMD assessment did not affect cardiovascular variables (HR, MAP, and P_ET_CO_2_, Table 1).

**Table 1:** Cardiopulmonary responses

	Time	2 h			4 h			6 h				*P* values		
	Inspirate	Eupnea	Hypercapnia	Δ	Eupnea	Hypercapnia	Δ	Eupnea	Hypercapnia	Δ		Time	Inspirate	Interaction
HR (bpm)	Normoxia	54 ± 13	53 ± 11	− 1 ± 2	57 ± 13	59 ± 12	2 ± 3	57 ± 10	60 ± 12	4 ± 6	Normoxia	0.019	0.138	0.126
	Hypoxia	62 ± 11	60 ± 10	− 3 ± 8	68 ± 19	68 ± 13	1 ± 11	72 ± 16	69 ± 12	− 3 ± 7	Hypoxia	0.035	0.577	0.868
											Δ	0.350	0.206	0.596
MAP (mmHg)	Normoxia	94 ± 12	99 ± 15	5 ± 5	93 ± 7	97 ± 5	4 ± 3	92 ± 7	95 ± 9	3 ± 5	Normoxia	0.830	0.069	0.906
	Hypoxia	84 ± 6	85 ± 3	2 ± 7	78 ± 9	78 ± 8	0 ± 6	81 ± 13	85 ± 12	4 ± 3	Hypoxia	0.097	0.420	0.806
											Δ	0.716	0.232	0.458
P_ET_CO_2_ (mmHg)	Normoxia	44 ± 7	51 ± 6	7 ± 2	44 ± 7	52 ± 6	8 ± 2	44 ± 3	51 ± 3	7 ± 1	Normoxia	0.634	< 0.001	0.952
	Hypoxia	37 ± 6	45 ± 3	8 ± 3	37 ± 5	44 ± 4	6 ± 3	33 ± 5	42 ± 4	9 ± 3	Hypoxia	0.010	< 0.001	0.387
											Δ	0.533	0.572	0.123

Values are mean ± SD

*HR* heart rate *MAP* mean arterial blood pressure, *P*_*ET*_*CO*_*2*_ end-tidal partial pressure of carbon dioxide

Bold p-values denote a P-value <0.05. The Bold has now been removed

### Blood flow

**Fig. 2:**
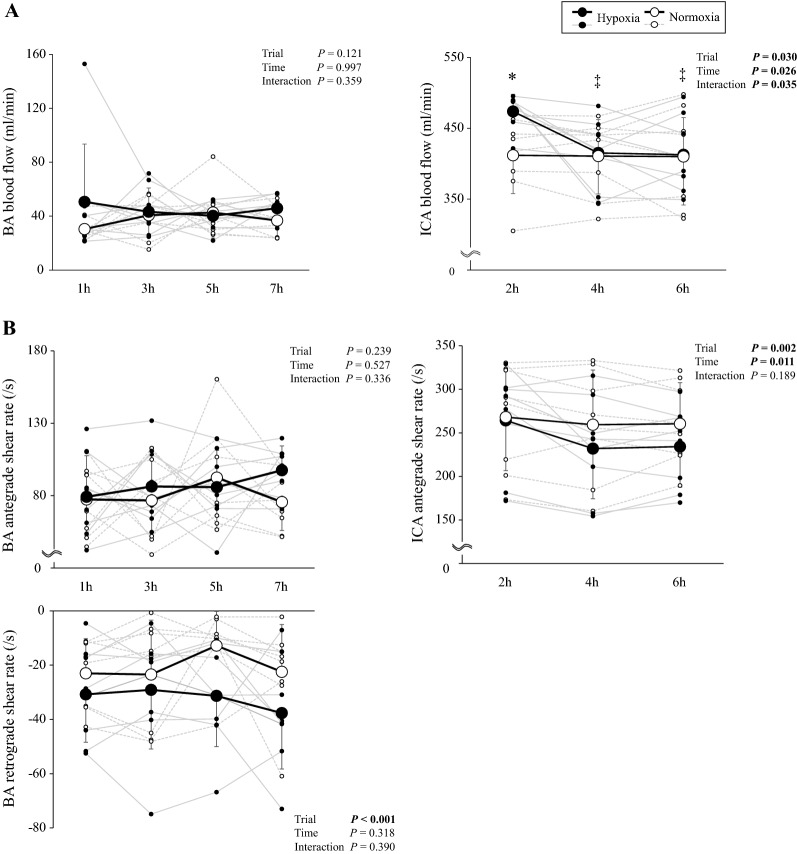
**A** Blood flow in brachial artery (BA) and internal carotid artery (ICA), and **B** antegrade shear rate (SR), retrograde SR in BA and antegrade SR in ICA (B) during hypoxia and normoxia. *n* = 8. Values are mean ± SD. ^*^P < 0.05 different vs. normoxia, ^‡^*P* < 0.05 different vs. 2 h

BA blood flow did not change during hypoxia (*P* = 0.359 *vs*. normoxia, Fig. 2A). ICA blood flow was higher at 2nd h during hypoxia than that of the normoxia condition (*P* = 0.001), while ICA blood flow did not differ between both conditions from 4^th^ h to the end of the hypoxia exposure.

Hypoxia did not alter BA antegrade SR (*P* = 0.336 *vs*. normoxia, Fig. 2B), whereas BA retrograde SR (absolute values) was consistently higher throughout (*P* < 0.001 *vs*. normoxia). In contrast, retrograde SR was not observed in the ICA, whereas ICA antegrade SR during hypoxia was lower than that of normoxia (*P* = 0.002 *vs*. normoxia).

### Systemic FMD

**Fig. 3:**
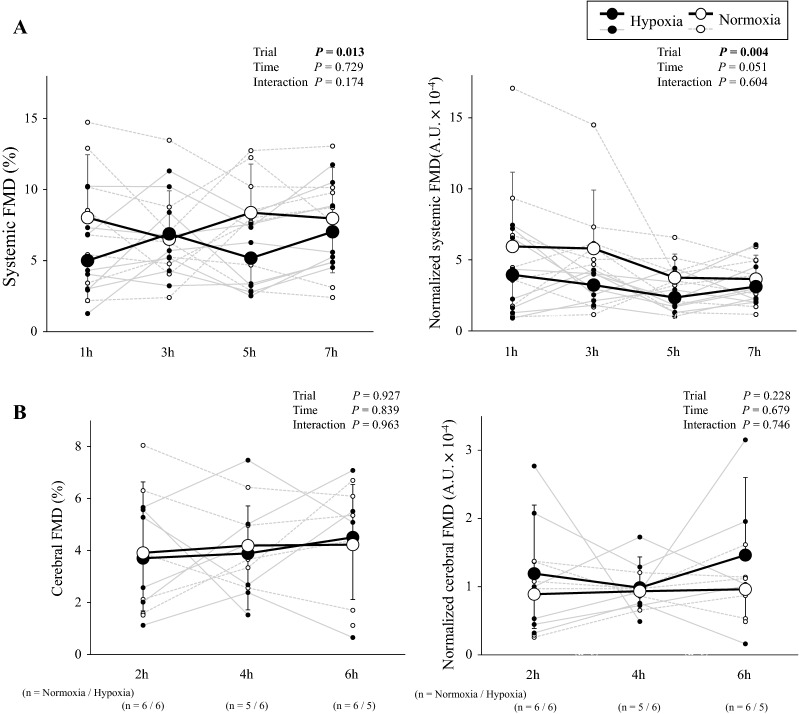
**A** Systemic (brachial artery, BA; *n* = 8) and **B** cerebral (internal carotid artery, ICA; *n* = 5 or 6) flow-mediated dilation (FMD) and normalized FMD during hypoxia and normoxia. Cerebral FMD sample size at each timepoint are in brackets along the horizontal axis. Values are mean ± SD

Hypoxia did not alter BA D_base_, D_peak_ or SR_AUC_ (*P* > 0.05 *vs*. normoxia; Table 2). Systemic FMD and normalized systemic FMD were consistently lower (*P* = 0.013 and *P* = 0.004 *vs*. normoxia, Fig. 3A). In addition, corrected systemic FMD remained lower during hypoxia (*P* = 0.053).

**Table 2:** Systemic flow-mediated dilation

Time	1 h		3 h			5 h		7 h		*P* values		
Trial	Normoxia	Hypoxia	Normoxia		Hypoxia	Normoxia	Hypoxia	Normoxia	Hypoxia	Time	Trial	Interaction
D_base_ (mm)	4.1 ± 0.3	4.6 ± 1.1	4.5 ± 0.8	4.4 ± 0.3		4.3 ± 0.4	4.3 ± 0.2	4.3 ± 0.3	4.3 ± 0.2	0.852	0.343	0.164
D_peak_ (mm)	4.4 ± 0.3	4.9 ± 1.1	4.8 ± 0.8	4.7 ± 0.3		4.6 ± 0.3	4.6 ± 0.3	4.7 ± 0.3	4.6 ± 0.2	0.885	0.715	0.311
ΔD (mm)	0.32 ± 0.16	0.22 ± 0.12	0.29 ± 0.15	0.30 ± 0.13		0.35 ± 0.13	0.22 ± 0.11	0.34 ± 0.15	0.30 ± 0.12	0.032	0.667	0.323
SR_AUC_ (a.u.)	18,224 ± 8798	15,520 ± 6645	14,776 ± 7421	21,835 ± 6802		23,244 ± 5618	23,019 ± 7191	22,277 ± 5658	23,700 ± 7648	0.016	0.405	0.209
Peak time (s)	153 ± 14	151 ± 21	152 ± 26	177 ± 97		183 ± 43	143 ± 25	151 ± 15	164 ± 33	0.858	0.819	0.159

Values are mean ± SD

*D*_*base*_ baseline diameter, *D*_*peak*_ peak diameter, *ΔD* the change in diameter from D_base_ to D_peak_, *SR*_*AUC*_ shear rate area under the curve from onset of hyperemia to peak dilation; Peak time, time to peak dilation from onset of hyperemia

Bold *p*-values denote a *p*-value less than P<0.05. The Bold has now been removed

### Cerebral FMD

**Fig. 4:**
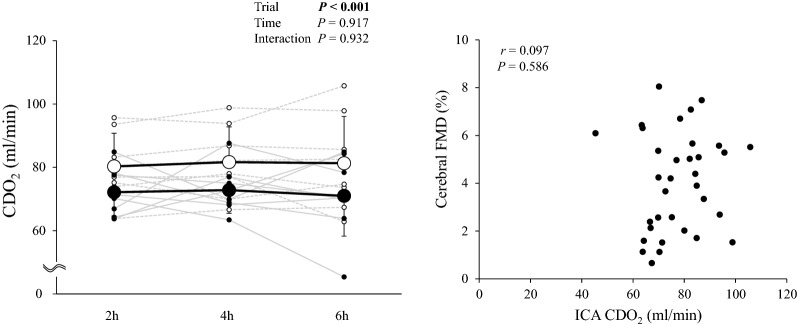
**A** Cerebral oxygen delivery (CDO_2_) and **B** relationship between CDO_2_ and cerebral FMD

Both ICA D_base_ and D_peak_ were slightly higher during hypoxia (*P* < 0.001 *vs*. normoxia), whereas in contrast, ICA SR_AUC_, cerebral FMD and normalized cerebral FMD were not altered (*P* = 0.927 and *P* = 0.228 *vs*. normoxia, Fig. 3B). In addition, corrected cerebral FMD remained unaltered during hypoxia (*P* = 0.480). While CDO_2_ was lower during hypoxia (*P* < 0.001 vs. normoxia), we failed to observe a relationship between CDO_2_ and cerebral FMD (*r* = 0.097, *P* = 0.586, Fig. 4).

## Discussion

Extending prior research highlighting the cerebrovascular benefits associated with cyclic intermittent hypoxia, the present study examined to what extent continuous steady-state exposure impacts the integrated SR and FMD responses in the systemic and cerebral circulation. Our primary finding is that while continuous hypoxia was associated with a reduction in systemic FMD, it failed to impact cerebral FMD despite a reduction in ICA antegrade SR. These findings contrast with those observed during cyclic intermittent hypoxia [7], highlighting the regulatory importance of (hypoxia) dose duration and flow/SR phenotype. Furthermore, the reduction in anterior CDO_2_ appears to be independent of local changes in EF.

### Systemic FMD

In the present study, systemic FMD was attenuated in hypoxia, whereas cerebral FMD remained preserved. The systemic FMD response to hypoxia remains controversial with some studies demonstrating a reduction [12–18], whereas others have failed to document any change [19–22]. These inconsistent findings may be related to differences in the duration and severity of hypoxia [16] and protocol including the hyperemic stimulus [12, 14, 15, 19, 21]. A recent study [16] reported that systemic FMD was reduced during 30 min of mild (S_a_O_2_ 93%) and moderate (S_a_O_2_ 83%) hypoxia. The reduction in systemic FMD was partly attributable to the observed (25%) reduction in SR_AUC_. In further support, Tremblay et al. [17] identified a 29% reduction in systemic FMD during acute hypoxia (20 min) and 25% reduction during sustained hypoxia (5–7 days) that correlated with changes in baseline BA mean and antegrade SR. However, these findings contrast with the present study given that hypoxia failed to alter BA antegrade SR, whereas the increase in BA retrograde SR was more marked and sustained. Therefore, our findings suggest that it is the increase in BA retrograde (*P* < 0.001, Fig. 2B) and not antegrade SR that underlies the hypoxia-induced reduction in systemic FMD (*P* = 0.239).

Interestingly, a reduction in systemic FMD during imposed oscillatory shear stress was found to be present even in normoxia, indicating that the shear stress phenotype may contribute to impaired vascular EF [17]. Indeed, the endothelium appears to be more susceptible to oscillatory shear stress during hypoxia, as the oscillatory shear stress intervention elicited an impairment in FMD during hypoxia but not normoxia combined with the observed dissociation between the change in SR_AUC_ and FMD [21]. Moreover, acute and progressive increases in baseline BA retrograde SR have been shown to elicit a dose-dependent impairment in systemic FMD [27]. Collectively, it is conceivable that alterations in the SR phenotype rather than hypoxia per se is the underlying stimulus regulating systemic FMD. The mechanism underlying the hypoxia-induced elevation in retrograde SR in the peripheral artery remains to be established. However, a previous study reported that classic sympathetic stimuli, such as lower body negative pressure increased retrograde flow and SR along with increased muscle sympathetic nerve activity (MSNA), indicating that activation of the sympathetic nerve (i.e., MSNA) may increase the retrograde flow and SR in the BA [39]. Thus, the hypoxia-induced elevation in retrograde SR may be related to a hypoxia-induced elevation in MSNA [39, 40].

### Cerebral FMD

In the present study, ICA blood flow increased at the 2nd h timepoint in hypoxia before returning to normoxic control values by the 4th h and thereonin (Fig. 2B). In contrast, no hypoxia-induced elevations were observed in BA flow (Fig. 2A) highlighting differential regulation across separate albeit functionally integrated vascular beds. While it was not our specific intent to focus on the precise mechanism(s) underlying this kinetic, it likely reflects some degree of initial ‘compensatory’ vasodilation to offset the reduction in arterial O_2_ content (CaO_2_) followed by (CaO_2_) ‘restoration’ facilitated by complex interactions between the respiratory and autonomic nervous systems, as indicated in our prior research highlighting progressive and antagonistic changes in the end-tidal partial pressure of oxygen (P_ET_O_2_) and MAP [11].

In contrast to systemic FMD, hypoxia failed to alter cerebral FMD and this remained well preserved. Thus, the attenuated CDO_2_ in the anterior cerebral circulation observed in our prior study [11], cannot be attributed to changes in local EF. Importantly, a dissociation between the systemic (reduction) and cerebral (preservation) FMD response has also been documented in young smokers [33]. These findings indicate that the mechanism(s) underlying vascular EF are clearly site-specific. Indeed, it is well established that compared to the systemic vasculature, the cerebrovasculature is more CO_2_ sensitive to provide tighter coupling of O_2_ delivery via increased perfusion given its disproportionately high(er) bioenergetic demands to support resting synaptic transmission. Equally, the cerebrovasculature needs to protect the blood–brain barrier from over-perfusion when the limits of autoregulation are potentially compromized [41].

One potential mechanism underlying the regional (FMD) heterogeneity during hypoxia may be due to the fact that retrograde SR is not observed in the cerebral circulation [8, 23, 24, 28]. Indeed, systemic FMD was reduced in the face of elevated retrograde SR during hypoxia. Previous studies [8, 28] have demonstrated that anterograde SR dominates in the cerebral circulation, yet the underlying mechanisms remain to be established. It is possible that the different hemodynamic properties between the cerebral and systemic vasculature may reflect the contrasting flow profiles to which these anatomically distinct but functionally integrated vascular beds are exposed [41]. In contrast, cerebral (ICA) antegrade SR was lower relative to normoxia with no measurable impact on the (cerebral) FMD response. These findings suggest that (lack of change in) retrograde SR is the primary stimulus underlying vascular EF. This reduction in cerebral antegrade SR may simply be the consequence of hypoxia-induced vasodilation (Table 3, ICA D_base_, *P* < 0.001), an evolutionarily conserved response that strives to maintain CDO_2_ in the face of arterial hypoxemia, albeit inadequate in the present study.

**Table 3:** Cerebral flow-mediated dilation

Time	2 h			4 h		6 h		*P* values		
Trial	Normoxia	Hypoxia		Normoxia	Hypoxia	Normoxia	Hypoxia	Time	Trial	Interaction
D_base_ (mm)	5.1 ± 0.7	5.3 ± 0.6	5.3 ± 0.7		5.4 ± 0.6	5.1 ± 0.6	5.2 ± 0.5	0.360	< 0.001	0.742
D_peak_ (mm)	5.3 ± 0.7	5.5 ± 0.7	5.5 ± 0.6		5.6 ± 0.6	5.3 ± 0.6	5.4 ± 0.6	0.451	< 0.001	0.724
ΔD (mm)	0.20 ± 0.13	0.20 ± 0.12	0.21 ± 0.06		0.20 ± 0.10	0.21 ± 0.12	0.23 ± 0.12	0.872	0.860	0.959
SR_AUC_ (a.u.)	47,490 ± 19,884	38,783 ± 15,567	45,474 ± 15,207		39,963 ± 14,721	41,379 ± 11,867	36,376 ± 10,821	0.413	0.059	0.972
Peak time (s)	148 ± 36	138 ± 27	148 ± 17		143 ± 25	141 ± 27	134 ± 37	0.784	0.454	0.983

Values are mean ± SD

*D*_*base*_ baseline diameter, *D*_*peak*_ peak diameter, *ΔD* the change in diameter from D_base_ to D_peak_, *SR*_*AUC*_ shear rate area under the curve from the onset of hyperemia to peak dilation; Peak time, time to peak dilation from the onset of hyperemia

Bold *p*-values denote a *p*-value less than P<0.05. The Bold has now been removed

Cerebral autoregulation can also modify the SR phenotype [42]. For example, continuous hypoxia decreases MAP [40, 43, 44] subsequent to hypoxia vasodilation and this can cause a reduction in cerebral antegrade SR subsequent to a decrease in blood flow velocity regardless of changes in blood flow. Another possible mechanism may be related to underlying differences between cerebral and systemic vasculature in the SR phenotype, the consequence of site-specific differences in anatomical/histological characteristics including redox status, hypoxia-induced sympathetic activation and the vascular territories they each subserve [23]. Since acute hypotension modifies systemic EF [31] hypoxia-induced hypotension may equally impact cerebral EF. The same concept applies for hypoxia-induced alterations in hemostasis and redox-status given their differential impact on systemic (and potentially cerebral) EF [15, 45, 46].

### Hypoxia stimulation related flow/SR phenotype for cerebral EF

Recently, the elevation in ICA antegrade SR during cyclic intermittent hypoxia has been reported [7]; however, the underlying mechanism remains unclear. The authors suggested that a cyclic intermittent hypoxia-induced elevation in cardiac output increases anterograde SR without elevating sympathetic activity (arterial blood pressure). In contrast, continuous hypoxia increases sympathetic activation and arterial blood pressure [47]. Thus, we speculate that the differential hemodynamic responses may contribute to the observed differences in cerebral FMD. Indeed, cyclic intermittent hypoxia was shown to improve cerebral FMD subsequent to a flow-mediated elevation in antegrade SR [7], in stark contrast to what we observed in the present study employing a continuous exposure paradigm. These findings suggest that oscillatory stress imposed by the ‘intermittency’ of the stimulus (e.g., high-intensity interval exercise and/or hypoxia) is the key stimulus underling vascular endothelial adaptation [48]. A previous study [49] clearly demonstrated that oscillatory shear stress improves EF in the systemic vasculature but this occurs via increased in both retrograde and anterograde SR. Some previous studies [17, 27, 50, 51] reported that experimentally induced oscillatory shear stress causes a transient reduction in systemic FMD, and demonstrate that an increase in retrograde SR contributes to this oscillatory shear stress-induced endothelial dysfunction. Importantly, retrograde SR is absent in the cerebral vasculature [28]. In the cerebral circulation, hypoxia stimulates oscillatory SR more markedly relative to continuous hypoxia conditions due to the absence of retrograde SR which serves to reduce (total) SR and consequently, it may enhance cerebral EF. However, the brain appears more sensitive to structural perturbation/damage (e.g., increased blood–brain barrier permeability) compared to the systemic vasculature given its increased bioenergetic demands and limited aerobic/glycolytic energy reserves [52].

A previous review proposed that hypoxic conditioning may be harmless and represent a promising adjunct therapy for stroke patients [53]. However, in the present study, continuous hypoxia’failed’ to improve cerebral FMD (i.e., it was simply maintained). In contrast, cyclic intermittent hypoxia may prove a useful non-pharmacological adjunct therapy for patients with brain disease (i.e., after the onset of stroke) given its impact on cerebral FMD is ‘superior’.

### Limitations

There are a number of limitations to the present study that warrant careful consideration. First, larger scale follow-up studies are encouraged to confirm our findings given the interpretive limitations associated with the relatively small sample sizes employed despite prospective adequate (prospective) powering of our study (albeit only against systemic FMD), including caveats associated with a Type M error [54]. While technical failure constrained our assessment of cerebral FMD to 5–6 participants, retrospective power calculations based on the observed effect size of 0.110 (calculated from partial *η*^2^ = 0.012), 1-*β* of 0.80, and *α* of 0.05, indicated that the sample size required to detect a treatment effect would be in excess of 200 participants, tentatively arguing against sample size inflation. Second, we chose to constrain our analyzes, focusing exclusively on men to (better) control for the potential vascular confounds caused by sex androgens [55]. There is an evolving body of literature suggesting that lifelong adaptation to hypoxia (phenotypical responses observed in native highlanders) confers neuroprotective benefits linked to more efficient redox-regulation of systemic [56, 57] and cerebrovascular [58] function and consequent O_2_ transport. It is conceivable that given such adaptations, highlanders may prove phenotypically less ‘responsive/sensitive’ to hypoxia although future studies are encouraged to better define the hypoxic dose stimulus (intensity/frequency/duration) and corresponding implications for integrated vascular endothelial function.

## Conclusions

Our findings demonstrate that continuous steady-state exposure to hypoxia was associated with a reduction in systemic FMD, yet failed to impact cerebral FMD despite a reduction in ICA antegrade SR. These findings contrast with those observed during cyclic intermittent hypoxia [7], highlighting the regulatory importance of (hypoxia) dose duration and flow/SR phenotype. Understanding the latter is key to designing interventions to optimize integrated systemic and cerebrovascular function in patients suffering from circulatory disease and consequent hypoxemia.

## Data Availability

The data sets used and analyzed during the current study are available from the corresponding author on reasonable request.
